# Supplementary material for: Perinatal thymic-derived CD8αβ-expressing γδ T cells are innate IFN-γ producers that expand in IL-7R–STAT5B-driven neoplasms
Source: Nat Immunol. 2024 May 27;25(7):1207–17. doi: 10.1038/s41590-024-01855-4 (PMC11224017; doi:10.1038/s41590-024-01855-4)
Supplement: Supplementary file 1 — Reporting Summary [file 41590_2024_1855_MOESM1_ESM.pdf]

nature portfolio

Corresponding author(s): Daniel J Pennington, Gina J Fiala

Last updated by author(s): Apr 19, 2024

Reporting Summary

Nature Portfolio wishes to improve the reproducibility of the work that we publish. This form provides structure for consistency and transparency in reporting. For further information on Nature Portfolio policies, see our Editorial Policies and the Editorial Policy Checklist.

Statistics

For all statistical analyses, confirm that the following items are present in the figure legend, table legend, main text, or Methods section.

n/a	Confirmed	
☐	☒	The exact sample size (*n*) for each experimental group/condition, given as a discrete number and unit of measurement
☐	☒	A statement on whether measurements were taken from distinct samples or whether the same sample was measured repeatedly
☐	☒	The statistical test(s) used AND whether they are one- or two-sided<br>*Only common tests should be described solely by name; describe more complex techniques in the Methods section.*
☐	☒	A description of all covariates tested
☒	☐	A description of any assumptions or corrections, such as tests of normality and adjustment for multiple comparisons
☐	☒	A full description of the statistical parameters including central tendency (e.g. means) or other basic estimates (e.g. regression coefficient) AND variation (e.g. standard deviation) or associated estimates of uncertainty (e.g. confidence intervals)
☐	☒	For null hypothesis testing, the test statistic (e.g. *F*, *t*, *r*) with confidence intervals, effect sizes, degrees of freedom and *P* value noted<br>*Give P values as exact values whenever suitable.*
☒	☐	For Bayesian analysis, information on the choice of priors and Markov chain Monte Carlo settings
☒	☐	For hierarchical and complex designs, identification of the appropriate level for tests and full reporting of outcomes
☒	☐	Estimates of effect sizes (e.g. Cohen's *d*, Pearson's *r*), indicating how they were calculated

Our web collection on statistics for biologists contains articles on many of the points above.

Software and code

Policy information about availability of computer code

Data collection	Flow cytometry data was acquired using FACSDiva v6.2 software (BD Bioscience).
Data analysis	All analysis are described in the relevant section of Methods.<br>Flow cytometry data was analysed using FlowJo software v10.6.1 or v10.8.1<br>Single-cell RNAseq data was analysed using the R package Seurat v5.0 and the Slingshot trajectory inference R package<br>Gene ontology and enrichment pathway analysis was performed using Metascape v3.5<br>human RNA-Seq data were aligned to the GRCh38 human genome reference by STAR1015<br>(version 2.7.11) and analysed using RSEM (version 1.3.0) with the batch correction by ComBat in the sva R1017<br>package; DESeq2 R package was used for the normalization of each gene expression.<br>Statistical analyses were done with GraphPad Prism v6.0 or v8.4.2

For manuscripts utilizing custom algorithms or software that are central to the research but not yet described in published literature, software must be made available to editors and reviewers. We strongly encourage code deposition in a community repository (e.g. GitHub). See the Nature Portfolio guidelines for submitting code & software for further information.

Data

Policy information about availability of data

All manuscripts must include a data availability statement. This statement should provide the following information, where applicable:

- Accession codes, unique identifiers, or web links for publicly available datasets
- A description of any restrictions on data availability
- For clinical datasets or third party data, please ensure that the statement adheres to our policy

The accession code for single cell RNA sequencing data is GSE167943. The data that support the findings of this study are available from the corresponding authors upon request.

Research involving human participants, their data, or biological material

Policy information about studies with human participants or human data. See also policy information about sex, gender (identity/presentation), and sexual orientation and race, ethnicity and racism.

Reporting on sex and gender	Sex and gender were not taken into account in the study design for human samples. Given the small number of gamma delta T-ALL patient samples that we could have access to, we used all that were available for this study. Nonetheless, we found that there was a male/female ratio of 2.08, which is within the known range reported in the literature for T-ALL.
Reporting on race, ethnicity, or other socially relevant groupings	We did not consider race, ethnicity or other socially relevant grouping in our analyses.
Population characteristics	Median age : 25 years; Age range : 4-70 years; Sex ratio (M/F): 2.08; Diagnosis: T-ALL.<br>Samples were collected at diagnosis, before treatment. We used 37 gamma-delta primary T-ALL samples and 5 PDX samples derived from primary T-ALL samples, previously collected at Necker Enfants-Malades Hospital (Paris, France). Mononuclear cells were isolated by ficoll density gradient and cryopreserved in liquid nitrogen.
Recruitment	We used cryopreserved samples from patients that were gamma-delta T-ALL, available at Necker Enfants-Malades Hospital (N=37+5).
Ethics oversight	All adult and pediatric cases were retrospectively selected from FRAALLE2000 and GRAALL2005 enrolled patients based on their TCRgd expressing immunophenotype and availability of frozen diagnostic material for CD8b staining. Pediatric cases were treated according to the FRALLE2000 T guidelines. Informed consent for data registration was provided according to the Declaration of Helsinki. This study was approved by the Leukemia's Committee of the National Scientific Committee of the SFCE (Société Française des Cancers de l'Enfant) and by the Ethics Committee of each participating center. Adults cases were enrolled within the multicenter randomized GRAAL-2005 protocol. Informed consent was obtained from all patients at trial entry. This study was conducted in accordance with the Declaration of Helsinki and approved by local and multicenter research ethical committees.

Note that full information on the approval of the study protocol must also be provided in the manuscript.

Field-specific reporting

Please select the one below that is the best fit for your research. If you are not sure, read the appropriate sections before making your selection.

☒ Life sciences ☐ Behavioural & social sciences ☐ Ecological, evolutionary & environmental sciences

For a reference copy of the document with all sections, see nature.com/documents/nr-reporting-summary-flat.pdf

Life sciences study design

All studies must disclose on these points even when the disclosure is negative.

Sample size	In a single experiment, at least three biological replicates were used. No sample size calculations were performed, but the sample sizes were determined based on our experimental observations and our experiences in giving reliable and reproducible results. Furthermore, data was collected in repeated independent experiments.
Data exclusions	Two biological replicates from neonatal small intestine analysis depicted in Fig. 2A were excluded due to absence of sufficient lymphocytes from the preparations.
Replication	At least three biological replicates were used for all experiments, except for one experiment of neonatal organs with two biological replicates pooled from 4 neonates each.<br>Experimental results depicted in Fig. 1B-C and Ext. Data Fig. 1B-C represent data from one experiment with four independent biological replicates.<br>Experimental results depicted in Fig. 1E-F, Ext. Data Fig. 1C, Fig. 2B, D, E, Ext. Data Fig.2C-D, Ext. Data Fig. 2F, Fig. 4D-E, Fig. 4G, Ext. Data Fig. 5B, Fig. 5A-F, and in Ext. Data Fig. 6A-G represent merged data from at least two independent experiments each.

Experimental results depicted in Fig. 2A summarizes eight (neonatal spleen), seven (neonatal LN), six (neonatal liver and adult spleen), five (adult LN), four (adult liver), three (neonatal lung) two (neonatal and adult intestine, neonatal kidney) and one experiments (adult lung and kidney).
Experimental results depicted in Fig. 2C, Ext. Data Fig. 2A, Fig. 3B-D, Ext. Data Fig. 3A, B, C, E, Fig. 4A-C, Fig. 4F, and Ext. Data Fig. 5A were independently repeated two times with similar results.
Experimental results in Ext. Data Fig. 2A depicts data from one experiment with three independent biological replicates originating from three to four pooled independent donors each. Experimental results in Ext. Data Fig. 2D, Ext. Data Fig. 3C, Ext. Data Fig. 4, and Ext. Data Fig. 5C depict data from one experiment each.
Experimental results depicted in Fig. 3A summarizes five experiments (neonatal and adult), three (young thymus) and two (embryonic thymus and old thymus), respectively.
Experimental results in Fig. 6 depict analyses of two patient cohort with 154 or 42 patients each.
Representative plots and graphs are presented and representative or pooled summaries shown. Descriptions of the number of replicates and independent experiments are included in the corresponding figure legends as well as information on the statistical tests performed and calculated P values.

Randomization

Randomization is not relevant to this study as samples in each experiment were treated uniformly and the same data analysis procedure was applied to all samples of the same experiment.

Blinding

Investigators were not blinded in this study because all results presented are based on quantitative analysis which is not subject to human biases.

Reporting for specific materials, systems and methods

We require information from authors about some types of materials, experimental systems and methods used in many studies. Here, indicate whether each material, system or method listed is relevant to your study. If you are not sure if a list item applies to your research, read the appropriate section before selecting a response.

Materials & experimental systems

n/a	Involved in the study
☐	☒ Antibodies
☒	☐ Eukaryotic cell lines
☒	☐ Palaeontology and archaeology
☐	☒ Animals and other organisms
☒	☐ Clinical data
☒	☐ Dual use research of concern
☒	☐ Plants

Methods

n/a	Involved in the study
☒	☐ ChIP-seq
☐	☒ Flow cytometry
☒	☐ MRI-based neuroimaging

Antibodies

Antibodies used

Antibody (clone), Fluorochrome Catalogue # Supplier

CD3 (17A2), BV650, 100229 Biolegend
CD3 (17A2), BV711, 100241 Biolegend
CD3e (145-2C11), unconjugated, 16-0031-85 eBioscience
CD4 (RM4-5), V500, 560782 BD
CD5 (53-7.3), PE, 553023 BD
CD8a (53-6.7), BUV805, 612898 BD
CD8a (53-6.7), BV711, 100759 Biolegend
CD8a (53-6.7), PE-Cy7, 100721 Biolegend
CD8a (53-6.7), PE, 100708 Biolegend
CD8a (53-6.7), AF700, 100730 Biolegend
CD8b (YTS156.7.7), BV421, 126629 Biolegend
CD8b (YTS156.7.7), FITC, 126606 Biolegend
CD8b (YTS156.7.7), APC, 126614 Biolegend
CD8b (H35-17.2), PerCP-Cy5.5, 46-0083-82 eBioscience
CD8b (SIDI8BEE), PE, 12-5273-42 eBioscience
CD8b (QA20A40), PE, 376703 Biolegend
CD11b (M1/70), FITC, 101205 Biolegend
CD11c (N418), FITC, 117306 Biolegend
CD16 (S17014E), FITC, 158007 Biolegend
CD19 (MB19-1), FITC, 101506 Biolegend
CD19 (6D5), biotin, 115504 Biolegend
CD24 (M1/69), Pacific Blue, 101820 Biolegend
CD27 (LG.3A10), PE-Dazzle594, 124228 Biolegend
CD28 (37.51), unconjugated, 16-0281-85 eBioscience
CD44 (IM7), PerCP-Cy5.5, 103032 Biolegend
CD44 (IM7), V500, 560780 BD
CD45 (30-F11), BV510, 103138 Biolegend
CD45RB (C363.16A), APC-Cy7, 103310 Biolegend

CD73 (TY/11.8), PE-Cy7, 25-0731-80 eBioscience
CD122 (TM-b1), PE, 12-1222-82 eBioscience
CD127 (IL-7Ra; SB/199), PE, 12-1273-82 eBioscience
Eomes (W17001A), A647, 157703 Biolegend
IFNg (XMG1.2), PE-Cy7, 25-7311-82 eBioscience
IL-17A (TC11-18H10.1), BV421, 506926 Biolegend
Ki67 (16A8), PE, 652404 Biolegend
Ly6A/E (D7), BV605, 108133 Biolegend
Ly6A/E (D7), APC, 108112 Biolegend
Ly6A/E (D7), PE-Cy7, 108114 Biolegend
MHCII (M5/114), FITC, 107606 Biolegend
NK1.1 (PK136), PE, 108708 Biolegend
NK1.1 (PK136), PE-Cy7, 108714 Biolegend
TCRb (H57-597), PerCP-Cy5.5, 109228 Biolegend
TCRb (H57-597), APC-Cy7, 109220 Biolegend
TCRb (H57-597), FITC, 109206 Biolegend
TCRb (H57-597), biotin, 109204 Biolegend
TCRd (GL3), FITC, 11-5711-85 eBioscience
TCRd (GL3), APC, 17-5711-82 eBioscience
TCRd (GL3), BV605, 118129 Biolegend
TNFa (MP6-XT22), APC, 506308 Biolegend
Vg1 (2.11), PE, 141106 Biolegend
Vg4 (UC3-10A6), FITC, 137704 Biolegend
Vg5 (536), PE, 137504 Biolegend
Vg7 (F2.67), FITC, kindly provided by Dr Pablo Pereira, Pasteur Institute, Paris, France
Vd4 (GL2), PE, 134905 Biolegend
Vd6 (C504.17C), FITC, 154807 Biolegend

Validation

The antibodies used in this study were used according to the manufacturer's recommendation. Validation was based on the description provided on the manufacturers' homepage.
Antibody (clone) Validation
CD3 (17A2) https://www.biolegend.com/en-gb/products/brilliant-violet-650-anti-mouse-cd3-antibody-7843?GroupID=BLG242
https://www.biolegend.com/en-gb/products/brilliant-violet-711-anti-mouse-cd3-antibody-10022
CD3e (145-2C11) https://www.thermofisher.com/antibody/product/CD3e-Antibody-clone-145-2C11-Monoclonal/14-0031-82
CD4 (RM4-5) https://www.bdbiosciences.com/en-us/products/reagents/flow-cytometry-reagents/research-reagents/single-color-antibodies-ruo/v500-rat-anti-mouse-cd4.560782
CD5 (53-7.3) https://www.bdbiosciences.com/en-us/products/reagents/flow-cytometry-reagents/research-reagents/single-color-antibodies-ruo/pe-rat-anti-mouse-cd5.553023
CD8a (53-6.7) https://www.bdbiosciences.com/en-gb/products/reagents/flow-cytometry-reagents/research-reagents/single-color-antibodies-ruo/buv805-rat-anti-mouse-cd8a.612898
https://www.biolegend.com/en-gb/products/alexa-fluor-700-anti-mouse-cd8a-antibody-3387
https://www.biolegend.com/de-de/products/brilliant-violet-711-anti-mouse-cd8a-antibody-7926?GroupID=BLG279
https://www.biolegend.com/en-gb/products/pe-cyanine7-anti-mouse-cd8a-antibody-1906?GroupID=BLG2559
https://www.biolegend.com/en-gb/products/pe-anti-mouse-cd8a-antibody-155?GroupID=BLG2559
CD8b (YTS156.7.7) https://www.biolegend.com/fr-lu/products/brilliant-violet-421-anti-mouse-cd8b-ly-3-antibody-17374
https://www.biolegend.com/en-gb/products/fitc-anti-mouse-cd8b-antibody-4475?GroupID=BLG4212
https://www.biolegend.com/en-gb/products/apc-anti-mouse-cd8b-antibody-9055
(H35-17.2) https://www.thermofisher.com/antibody/product/CD8b-Antibody-clone-eBioH35-17-2-H35-17-2-Monoclonal/46-0083-82
CD11b (M1/70) https://www.biolegend.com/en-gb/products/fitc-anti-mouse-human-cd11b-antibody-347
CD11c (N418) https://www.biolegend.com/en-gb/products/fitc-anti-mouse-cd11c-antibody-1815?GroupID=BLG11937
CD16 (S17014E) https://www.biolegend.com/en-gb/sean-tuckers-tests/fitc-anti-mouse-cd16-antibody-19303?GroupID=ImportedGROUP1
CD19 (MB19-1) https://www.biolegend.com/en-gb/sean-tuckers-tests/fitc-anti-mouse-cd19-antibody-1971?GroupID=BLG4752
CD19 (6D5) https://www.biolegend.com/en-ie/products/biotin-anti-mouse-cd19-antibody-1527?GroupID=BLG7045
CD24 (M1/69) https://www.biolegend.com/en-gb/products/pacific-blue-anti-mouse-cd24-antibody-3584
CD27 (LG.3A10) https://www.biolegend.com/en-ie/products/pe-dazzle-594-anti-mouse-rat-human-cd27-antibody-11906
CD28 (37.51) https://www.thermofisher.com/antibody/product/CD28-Antibody-clone-37-51-Monoclonal/16-0281-82
CD44 (IM7) https://www.bdbiosciences.com/en-nz/products/reagents/flow-cytometry-reagents/research-reagents/single-color-antibodies-ruo/v500-rat-anti-mouse-cd44-pgp-1-ly-24.560780
CD45 (30-F11) https://www.biolegend.com/ja-jp/products/brilliant-violet-510-anti-mouse-cd45-antibody-7995?GroupID=BLG1932
CD45RB (C363.16A) https://www.biolegend.com/en-gb/products/apc-cyanine7-anti-mouse-cd45rb-antibody-3525?GroupID=BLG259
CD73 (TY/11.8) https://www.thermofisher.com/antibody/product/CD73-Antibody-clone-eBioTY-11-8-TY-11-8-Monoclonal/25-0731-82
CD122 (TM-b1) https://www.thermofisher.com/antibody/product/CD122-Antibody-clone-TM-b1-TM-beta1-Monoclonal/12-1222-82
CD127 (SB/199) https://www.thermofisher.com/antibody/product/CD127-Antibody-clone-eBioSB-199-SB-199-Monoclonal/12-1273-82
Eomes (W17001A) https://www.biolegend.com/en-gb/products/alexa-fluor-647-anti-mouse-eomes-antibody-18078
IFNg (XMG1.2) https://www.thermofisher.com/antibody/product/IFN-gamma-Antibody-clone-XMG1-2-Monoclonal/25-7311-82
IL-17A (TC11-18H10.1) https://www.biolegend.com/en-gb/products/brilliant-violet-421-anti-mouse-il-17a-antibody-7223
Ki67 (16A8) https://www.biolegend.com/en-gb/products/pe-anti-mouse-ki-67-antibody-8134?GroupID=GROUP26
Ly6A/E (D7) https://www.biolegend.com/en-gb/products/brilliant-violet-605-anti-mouse-ly-6a-e-sca-1-antibody-8664
https://www.biolegend.com/en-gb/products/apc-anti-mouse-ly-6a-e-sca-1-antibody-225
https://www.biolegend.com/en-gb/products/pe-cyanine7-anti-mouse-ly-6a-e-sca-1-antibody-3137
MHCII (M5/114) https://www.biolegend.com/en-gb/products/fitc-anti-mouse-i-a-i-e-antibody-366
NK1.1 (PK136) https://www.biolegend.com/en-gb/products/pe-anti-mouse-nk-1-1-antibody-431
https://www.biolegend.com/en-gb/products/pe-cyanine7-anti-mouse-nk-1-1-antibody-2840

TCRb(H57-597) https://www.biolegend.com/en-gb/products/percp-cyanine5-5-anti-mouse-tcr-beta-chain-antibody-5603
https://www.biolegend.com/en-ie/products/apc-cyanine7-anti-mouse-tcr-beta-chain-antibody-4137?GroupID=BLG6994
https://www.biolegend.com/en-gb/products/fitc-anti-mouse-tcr-beta-chain-antibody-270
https://www.biolegend.com/en-ie/products/biotin-anti-mouse-tcr-beta-chain-antibody-269
TCRd (GL3) https://www.thermofisher.com/antibody/product/TCR-gamma-delta-Antibody-clone-eBioGL3-GL-3-GL3-Monoclonal/11-5711-85
https://www.thermofisher.com/antibody/product/TCR-gamma-delta-Antibody-clone-eBioGL3-GL-3-GL3-Monoclonal/17-5711-82
https://www.biolegend.com/en-gb/products/brilliant-violet-605-anti-mouse-tcr-gamma-delta-antibody-9655
TNFa (MP6-XT22) https://www.biolegend.com/en-gb/products/apc-anti-mouse-tnf-alpha-antibody-975?GroupID=GROUP24
Vg1 (2.11) https://www.biolegend.com/en-gb/products/pe-anti-mouse-tcr-vgamma1-1-cr4-antibody-7039
Vg4 (UC3-10A6) https://www.biolegend.com/en-gb/products/fitc-anti-mouse-tcr-vgamma2-antibody-6536
Vg5 (536) https://www.biolegend.com/en-gb/products/pe-anti-mouse-tcr-vgamma3-antibody-6525
Vg7 (F2.67) https://www.biolegend.com/en-gb/products/purified-anti-mouse-tcr-vgamma7-antibody-20318
Vd4 (GL2) https://www.biolegend.com/en-gb/products/pe-anti-mouse-tcr-vdelta4-antibody-6105
Vd6 (C504.17C) https://www.biolegend.com/en-gb/products/fitc-anti-mouse-tcr-vdelta6-3-antibody-16294

Animals and other research organisms

Policy information about studies involving animals; ARRIVE guidelines recommended for reporting animal research, and Sex and Gender in Research

Laboratory animals

C57BL/6 wild-type (B6 WT), PI3Kδ-deficient mice (p110d-/-), PI3Kδ-hyperactive mice (p110dE1020K), Rorc(gt)-GfpTG reporter mice (RORgt-GFP+/-), TCRa-deficient mice (TCRa-/-), TCRb-deficient mice (TCRb-/-), and b2m-deficient mice (b2m-/-), Rag2-/-gc-/- mice, IL-4R-/- mice, Rosa26-hIL-7R.huCD2-Cre mice, hSTAT5BN642H mice (official name: C57BL/6N-Tg(STAT5B<N642H>)726Biat). All strains were on a C57BL/6 background. Mice were foetal (E15-E17), neonatal (2-4 days), young (10-11 days), adult (4-14 weeks) or aged (31-39 weeks). Embryos were from timed pregnancies or in vitro fertilization. Both male and female animals were used in this study and matched with controls of corresponding age and sex.

Wild animals

The study does not involve wild animals.

Reporting on sex

Results presented include both female and male animals. Sex was determined phenotypically whenever possible. No information on embryo sex was collected. For young and old animals sex of individuals was determined but no sex-specific effects were observed in the tissues analysed. In a limited set of neonates sex was determined but no sex-specific effects observed in the tissues analysed (thymus, spleen LN and liver). In adult mice, there might be a tendency of increased percentages of CD8ab gd T cells in male thymus, LN and spleen compared to females. A detailed analysis has not been performed. Data on other tissues (lungs, kidneys and small intestine) originates from female animals only.

Field-collected samples

The study did not involve field-collected samples.

Ethics oversight

All experiments involving animals were approved by the respective institutional ethics committees and performed in full compliance with UK Home Office and Portugal's Direção-Geral da Alimentação e Veterinária regulations and institutional guidelines.
Breeding and in vitro fertilization of C57BL/6N-Tg(STAT5B<N642H>)726Biat mice was approved by the institutional ethics committees of University of Veterinary Medicine Vienna and the Champalimaud Centre for the Unknown (Lisbon, Portugal).

Note that full information on the approval of the study protocol must also be provided in the manuscript.

Plants

Seed stocks

n.a.

Novel plant genotypes

n.a.

Authentication

n.a.

Flow Cytometry

Plots

Confirm that:

☒ The axis labels state the marker and fluorochrome used (e.g. CD4-FITC).

☒ The axis scales are clearly visible. Include numbers along axes only for bottom left plot of group (a 'group' is an analysis of identical markers).

☒ All plots are contour plots with outliers or pseudocolor plots.

☒ A numerical value for number of cells or percentage (with statistics) is provided.

Methodology

Sample preparation	Tumours were processed using the mouse-specific tumour dissociation kit (Miltenyi Biotec), according to the manufacturer's instructions. Briefly, tumours were chopped into small pieces before dissociation using a heat and enzyme-assisted program on the gentleMACS dissociator (Miltenyi Biotec). Subsequently, cell suspensions were filtered through 70 um cell strainers, red blood cells were lysed, and lymphocytes were enriched following Percoll (Sigma-Aldrich) density centrifugation. Single-cell suspensions of foetal thymocytes were obtained by gently homogenizing thymic lobes followed by straining through a 30 um nylon gauze (Sefar Ltd., UK) or a 40 um cell strainer. To obtain single-cell suspensions of lymphocytes from adult mice, peripheral lymph nodes (axillary and inguinal), thymus and spleen were dissected and strained through a 100 um cell strainer. Livers, lungs and kidneys were dissected and cut into pieces. Small intestines were dissected, flushed with ice-cold PBS, cut open longitudinally and into pieces. Organ pieces were digested in RPMI supplemented with 10% FBS containing 1 mg/ml collagenase type IV (Roche) and 100 ug/ml DNase I (Sigma) for 30 min shaking at 37°C, followed by filtering through an 100 um cell strainer. Cells were resuspended in a 40% isotonic Percoll solution and centrifuged on a 80% Percoll solution for 20 min at 700 x g at room temperature with brake off. Leukocytes were recovered from the interface, resuspended, and used for further analyses. Erythrocytes from blood, spleen, liver, lung and kidney samples were osmotically lysed in ACK lysis buffer (Invitrogen), and cells were washed in FACS buffer. Rosa26-hIL-7R.huCD2-Cre leukaemic thymus and lymph node cells were isolated as described, homogenized in HI-FBS containing 10% dimethylsulfoxide (DMSO) and frozen at -80 °C until used. Samples were then thawed, washed and homogenized in complete RPMI-1640.
Instrument	Sorts were performed on FACS AriaII and FACS AriaIII cell sorters (BD). Samples were acquired using an LSR-II and LSRFortessa X-20 flow cytometer (BD) or Canto II (BD)
Software	Analysis of Flow Cytometry data was performed using FlowJo.
Cell population abundance	Post-sort purity of gd T cell populations from pooled adult LN and spleen of WT or TCRa-/- mice was determined by re-run and recording of aliquots of purified populations on the flow cytometer. Analysis of re-run data acquired confirmed purities of from >90% to >99%.
Gating strategy	The gating strategy to determine abundance of CD8-expressing gd T cell subsets within organs and FTOC was the following: 1. SSC-A/time, 2. lymphocytes gate by FSC-A/SSC-A, 3. single cell gating and doublet discrimination (FSC-A/FSC-H and SSC-A/SSC-H), 4. live vs dead cells, 5. gating on gd T cells (either CD3+/TCRd+ or TCRd+/TCRb- or TCRd+/SSC), 6. CD8a/CD8b or Ly6a/CD8b. The gating strategy is displayed as Extended Data Fig. 1A.

☒ Tick this box to confirm that a figure exemplifying the gating strategy is provided in the Supplementary Information.
